# The Role of TRP Channels and PMCA in Brain Disorders: Intracellular Calcium and pH Homeostasis

**DOI:** 10.3389/fcell.2021.584388

**Published:** 2021-01-28

**Authors:** Sung-Min Hwang, Ji Yeon Lee, Chul-Kyu Park, Yong Ho Kim

**Affiliations:** ^1^Gachon Pain Center, Department of Physiology, Gachon University College of Medicine, Incheon, South Korea; ^2^Gil Medical Center, Department of Anesthesiology and Pain Medicine, Gachon University, Incheon, South Korea

**Keywords:** TRP channels, brain pathology, neurodegenerative diseases, calcium, pH, homeostasis, neuron

## Abstract

Brain disorders include neurodegenerative diseases (NDs) with different conditions that primarily affect the neurons and glia in the brain. However, the risk factors and pathophysiological mechanisms of NDs have not been fully elucidated. Homeostasis of intracellular Ca^2+^ concentration and intracellular pH (pH_i_) is crucial for cell function. The regulatory processes of these ionic mechanisms may be absent or excessive in pathological conditions, leading to a loss of cell death in distinct regions of ND patients. Herein, we review the potential involvement of transient receptor potential (TRP) channels in NDs, where disrupted Ca^2+^ homeostasis leads to cell death. The capability of TRP channels to restore or excite the cell through Ca^2+^ regulation depending on the level of plasma membrane Ca^2+^ ATPase (PMCA) activity is discussed in detail. As PMCA simultaneously affects intracellular Ca^2+^ regulation as well as pH_i_, TRP channels and PMCA thus play vital roles in modulating ionic homeostasis in various cell types or specific regions of the brain where the TRP channels and PMCA are expressed. For this reason, the dysfunction of TRP channels and/or PMCA under pathological conditions disrupts neuronal homeostasis due to abnormal Ca^2+^ and pH levels in the brain, resulting in various NDs. This review addresses the function of TRP channels and PMCA in controlling intracellular Ca^2+^ and pH, which may provide novel targets for treating NDs.

## Introduction

Calcium (Ca^2+^) is a second messenger involved in numerous signal transduction pathways, including cell proliferation, cell growth, neuronal excitability, metabolism, apoptosis, and differentiation (Berridge et al., 2000; Gleichmann and Mattson, 2011; Maklad et al., 2019). Intracellular Ca^2+^ has a complex role in brain signaling and regulates brain physiology to maintain neuronal integrity (Marambaud et al., 2009; Bezprozvanny, 2010; Kawamoto et al., 2012). Ca^2+^ influx across the plasma membrane is important for fundamental brain functions which are mainly mediated by glutamate receptor channels, voltage-gated Ca^2+^ channels, sodium-calcium exchanger, and transient receptor potential (TRP) channels (Bezprozvanny, 2010; Cross et al., 2010; Gees et al., 2010; Cuomo et al., 2015; Kumar et al., 2016). Thus, Ca^2+^ signaling affects a variety of neuronal functions in diverse physiological roles, and Ca^2+^ must be tightly regulated to avoid uncontrolled responses that can lead to pathological conditions (Kumar et al., 2016). However, sustained increase in Ca^2+^ influx induces endoplasmic reticulum stress, mitochondrial dysfunction, and various proteases, resulting in neuronal cell death (Bezprozvanny, 2010; Kawamoto et al., 2012). Indeed, impaired cell function caused by reactive nitrogen (oxygen) species and abnormal pH homeostasis also underpins the pathophysiology of neurodegenerative diseases (NDs) (Piacentini et al., 2008; Bezprozvanny, 2010; Gleichmann and Mattson, 2011; Zundorf and Reiser, 2011; Harguindey et al., 2017, 2019; Popugaeva et al., 2017). In particular, the maintenance of Ca^2+^ and pH levels is involved in a variety of NDs, including Alzheimer's disease (AD), Parkinson's disease (PD), Huntington's disease (HD), amyotrophic lateral sclerosis (ALS), and age-related disorders (Harguindey et al., 2007; Kumar et al., 2009; Smaili et al., 2009; Ruffin et al., 2014; Hong et al., 2020; Thapak et al., 2020). Extensive literature indicates that an excessive increase in cytosolic Ca^2+^ and H^+^ constitutes both direct and indirect ND-induced processes (Marambaud et al., 2009; Smaili et al., 2009; Bezprozvanny, 2010; Ruffin et al., 2014; Zhao et al., 2016; Harguindey et al., 2017).

TRP channels constitute a large family of membrane Ca^2+^ channels involved in a wide range of processes including thermoregulation, osmosis, pH, stretch, and chemical signaling (Kaneko and Szallasi, 2014). Functionally, activation of TRP channels influences Ca^2+^ signaling by allowing Ca^2+^ to enter the cell (cell depolarization), which may activate voltage-gated Ca^2+^ channels (Nilius and Owsianik, 2011; Vennekens et al., 2012). TRP channels in neuronal cells regulate voltage-gated Ca^2+^, K^+^, and Na^+^ channels, whereas TRP channel regulation in glial cells results in reduced Ca^2+^ entry via ORAI by membrane depolarization, or increased Ca^2+^ influx through the hyperpolarization of the membrane (Gees et al., 2010). In the central nervous system, TRP channels are widely expressed throughout the brain and play an essential role in regulating Ca^2+^ homeostasis associated with various cellular functions, including synaptic plasticity, synaptogenesis, and synaptic transmission in a specific region of the brain (Venkatachalam and Montell, 2007; Kaneko and Szallasi, 2014; Jardin et al., 2017; Chi et al., 2018; Hong et al., 2020). In addition, TRP subtype channels are expressed simultaneously or separately in neurons and glia, fulfilling critical roles in cell homeostasis, development, neurogenesis, and synaptic plasticity (Vennekens et al., 2012). Several members of the TRP subtype are highly expressed in neurons and glia (Moran et al., 2004; Butenko et al., 2012; Ho et al., 2014; Ronco et al., 2014; Verkhratsky et al., 2014; Liu et al., 2017; Rakers et al., 2017) (Table 1). Thus, diverse TRP channels expressed in the brain are involved in the progression of NDs such as Parkinson's and Alzheimer's. In particular, increased intracellular Ca^2+^ via TRP channels contributes to various pathophysiological events (Venkatachalam and Montell, 2007; Kaneko and Szallasi, 2014; Moran, 2018; Hong et al., 2020) as well as brain disorders such as AD, PD, stroke, epilepsy, and migraine (Table 1)(Morelli et al., 2013; Kaneko and Szallasi, 2014; Kumar et al., 2016; Moran, 2018; Hong et al., 2020; Liu et al., 2020).

**Table 1:** A summary of the transient receptor potential (TRP) subtypes found in distribution of central nervous system (CNS) cell types.

**TRP channels**		**Expression in brain**	**Expression in glia**	**Disorders**	**References**
TRPC subfamily	TRPC1	- Cerebellum, hippocampus, forebrain - Dopaminergic neuron (Human/mouse)	Astrocyte, microglia,	NDs, ADs, PD, HD,	Riccio et al., 2002; Bollimuntha et al., 2005, 2006; Selvaraj et al., 2009, 2012; Hong et al., 2015
	TRPC3	- Cerebellum, hippocampus, forebrain - Dopaminergic neuron (Human)	Astrocyte,	NDs, ADs, PDs	Rosker et al., 2004; Wu et al., 2004; Yamamoto et al., 2007; Mizoguchi et al., 2014
	TRPC4	Cerebellum, hippocampus, forebrain	Astrocyte,	Epilepsy	Wang et al., 2007; Wu et al., 2008; Von Spiczak et al., 2010; Tai et al., 2011
	TRPC5	- Cerebellum, forebrain - Hippocampus (mouse)	Astrocyte,	NDs, PDs, Epilepsy	Shin et al., 2010; Tai et al., 2011; Kaczmarek et al., 2012
	TRPC6	Cerebellum, hippocampus, forebrain, striatum	Astrocyte, microglia	NDs, ADs	Lessard et al., 2005; Wang et al., 2015; Liu et al., 2017; Lu et al., 2017
TRPM subfamily	TRPM2	- Hippocampus, forebrain - Cerebellum (human), cortex (rat)	Astrocyte, microglia	NDs, ADs, PDs	Fonfria et al., 2005; Kaneko et al., 2006; Hermosura et al., 2008; Ostapchenko et al., 2015
	TRPM7	- Cerebellum, forebrain, - Hippocampus (human) - cortex (mouse)	Astrocyte, microglia	NDs, ADs, PDs, Epilepsy	Aarts and Tymianski, 2005; Hermosura et al., 2005; Chen X. et al., 2010; Coombes et al., 2011; Oakes et al., 2019
TRPV subfamily	TRPV1	- Basal ganglia, hindbrain Cerebellum - Hippocampus (rat/mouse),	Astrocyte, microglia	NDs, AD, HD, epilepsy	Lastres-Becker et al., 2003; Kim et al., 2005; Gibson et al., 2008; Li et al., 2008; Lee et al., 2011; Balleza-Tapia et al., 2018
	TRPV4	Cerebellum, hippocampus,	Astrocyte, microglia	NDs, AD,	Auer-Grumbach et al., 2010; Chen D. H. et al., 2010; Landoure et al., 2010; Klein et al., 2011; Wang et al., 2019
TRPA subfamily	TRPA1	Cerebellum, hippocampus,	Astrocyte, oligodendrocyte	AD	Shigetomi et al., 2011; Lee et al., 2016; Saghy et al., 2016; Bolcskei et al., 2018

*PMCA, plasma membrane Ca^2+^ ATPase; AD, Alzheimer's disease; PD, Parkinson's disease; ND, neurodegenerative disease*.

**Figure 1:**
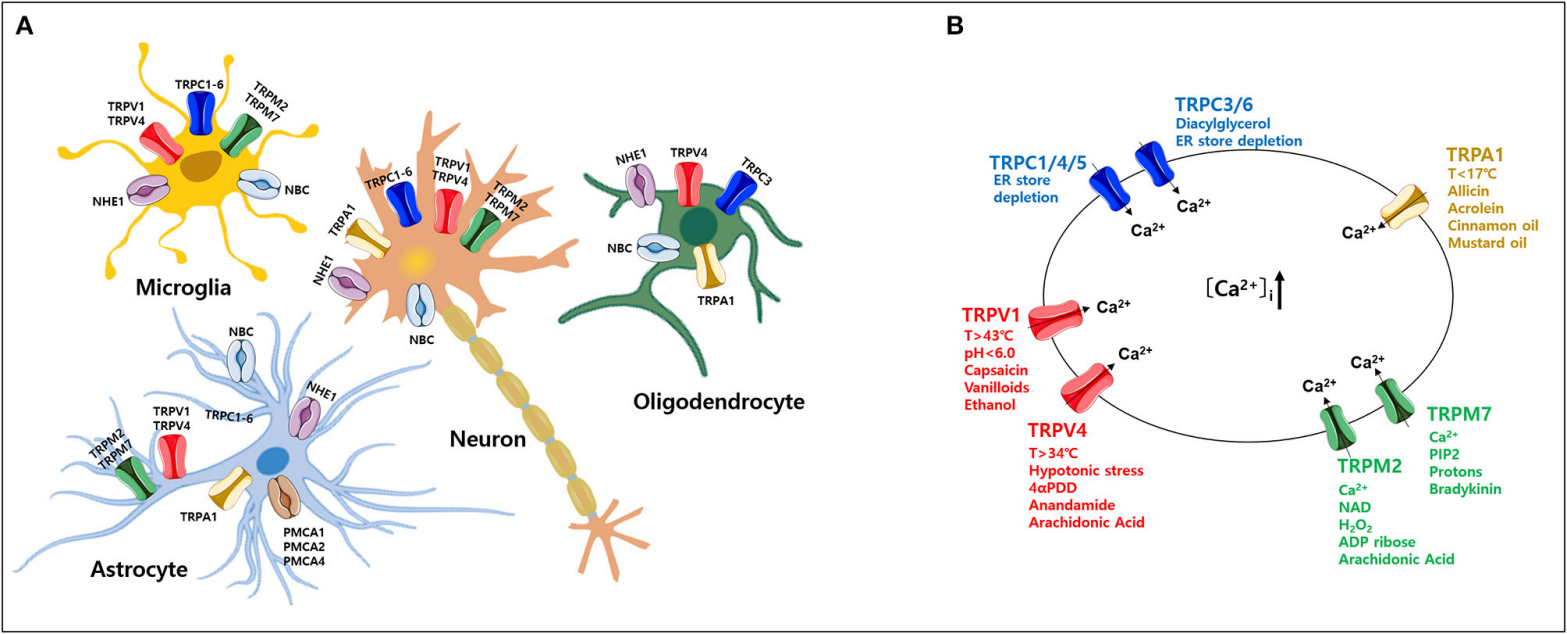
Expression of various transient receptor potential (TRP) subtypes and calcium (Ca^2+^) influx by their agonists in the mammalian central nervous system (CNS). **(A)** Expression profile of various TRP channels, NHE1, and NBC, in mammalian CNS cell types. **(B)** Ca^2+^ influx through activation of TRP subtypes by various agonists or activators in the mammalian CNS. TRP, transient receptor potential; PMCA, plasma membrane Ca^2+^ ATPase; NBC, Na^+^/${\text{HCO}}_{3}^{-}$ cotransporters; NHE, Na^+^/H^+^ exchangers.

**Figure 2:**
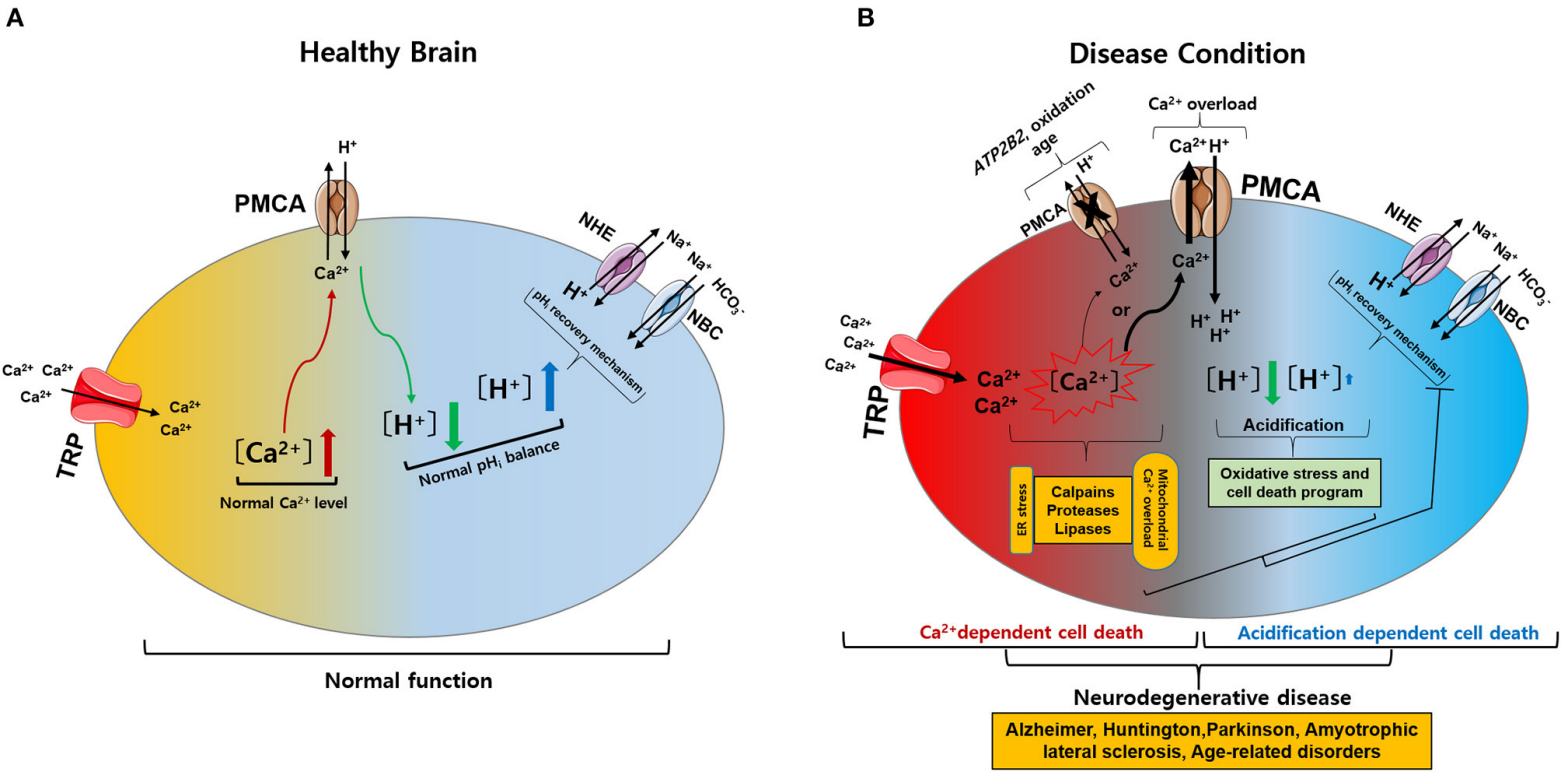
Intracellular calcium (Ca^2+^) and pH (pH_i_) signaling by activation of TRP and PMCA in healthy and diseased condition of the brain. **(A)** Normal physiological function of intracellular Ca^2+^ and pH_i_ homeostasis. The activation of TRP channels leads to Ca^2+^ influx into the cytosol. Increased Ca^2+^ levels are regulated by PMCA. The activation of PMCA can cause acidification. Acidification conditions are mediated by pH_i_ recovery functions regulated by NBC and NHE. **(B)** Neurodegenerative diseases caused by pathophysiological functions of intracellular Ca^2+^ and pH_i_ homeostasis. (1) The activation of TRP channels leads to excess Ca^2+^ influx and overload Ca^2+^ is maintained due to *ATP2B2*, oxidation, and age-related downregulation of PMCA: Ca^2+^-dependent cell death. (2) PMCA overexpression due to cytoplasmic Ca^2+^ overload cause persistent acidification from inhibition of the pH_i_ recovery mechanism by oxidative stress or cell death program: acidification dependent cell death. Ultimately, abnormal intracellular Ca^2+^ and pH_i_ levels impair neuronal function, resulting in neurodegenerative diseases. TRP, transient receptor potential; PMCA, plasma membrane Ca^2+^ ATPase; NBC, Na^+^/${\text{HCO}}_{3}^{-}$ cotransporters; NHE, Na^+^/H^+^ exchangers.

The normal regulation of intracellular Ca^2+^ levels involves mechanisms that control the specific uptake and extrusion mechanisms across the cell membrane (Kawamoto et al., 2012; Strehler and Thayer, 2018). Ca^2+^ influx is mediated by several voltage- and ligand-gated channels as well as transporters. Conversely, Ca^2+^ extrusion is dependent on Ca^2+^ pumps and Na^+^/Ca^2+^ exchangers (Strehler and Thayer, 2018). Among these, plasma membrane Ca^2+^ ATPases (PMCAs) actively extrude Ca^2+^ ions out of cells (Boczek et al., 2019). Thus, these pumps are important gatekeepers for maintaining intracellular Ca^2+^ homeostasis in cells (Stafford et al., 2017; Boczek et al., 2019). However, PMCA dysfunction causes altered Ca^2+^ homeostasis and leads to a persistent increase in cytosolic Ca^2+^, which can be neurotoxic and can accelerate the development of NDs and cognitive impairments as the person ages (Strehler and Thayer, 2018; Boczek et al., 2019). In particular, it is possible that the regulation of Ca^2+^ concentration might be more sensitive in which the cells are expressed both TRP and PMCA in the particular brain region (Figure 1). Thereby, abnormal expression of either TRP or PMCA subtype may be more likely to cause ND than other parts of the brain (Figure 2) (Minke, 2006; Stafford et al., 2017). In addition, PMCA activity is associated with intracellular acidification (Hwang et al., 2011) which is associated with neurological conditions observed among AD patients and other ND patients (Kato et al., 1998; Hamakawa et al., 2004; Mandal et al., 2012; Ruffin et al., 2014; Tyrtyshnaia et al., 2016).

It is crucial to investigate whether increased Ca^2+^ and (or) acidification are risk factors that affects ND-induced processes (Chesler, 2003; Hwang et al., 2011; Ruffin et al., 2014; Cuomo et al., 2015; Stafford et al., 2017; Boczek et al., 2019). Here, we review the involvement of TRP channels and PMCA in the pathophysiology of NDs.

## Brain Disorders

### Neurodegenerative Diseases

NDs such as AD, PD, HD, and ALS are age-related conditions characterized by uncontrolled neuronal death in the brain (Hong et al., 2020; Slanzi et al., 2020; Thapak et al., 2020). To date, several studies have reported that NDs are associated with protein aggregation, oxidative stress, inflammation, and abnormal Ca^2+^ homeostasis (Sprenkle et al., 2017). The impairment of Ca^2+^ homeostasis is known to result in increased susceptibility to NDs (Kumar et al., 2009; Smaili et al., 2009; Bezprozvanny, 2010; Gleichmann and Mattson, 2011; Kawamoto et al., 2012; Bagur and Hajnoczky, 2017). In particular, this impairment is associated with changes in Ca^2+^ buffering capacity, deregulation of Ca^2+^ channel activity, and alteration in other calcium regulatory proteins that occur in some types of neurons and glial cells in certain brain regions (Zundorf and Reiser, 2011; Nikoletopoulou and Tavernarakis, 2012). There is also increased Ca^2+^ influx mediated by abnormal TRP channel activation (Sawamura et al., 2017). Similarly, Ca^2+^ extrusion through PMCA has been shown to decrease in aged neurons (Jiang et al., 2012). For this reason, these NDs are associated with Ca^2+^ channels in neurons and glial cells (astrocytes, microglia, and oligodendrocytes), which are important for neuronal survival, myelin formation, neuronal support, and regulation of local neuron activity (neurons-glial signaling) (Zhang and Liao, 2015; Cornillot et al., 2019; Enders et al., 2020).

### Pathophysiological Role of TRP Channels

TRP channels are non-selective, Ca^2+^-permeable channels that regulate diverse cellular functions in neurons (Nilius, 2007; Venkatachalam and Montell, 2007; Sawamura et al., 2017). Based on functional characterization of TRP channels by a wide range of stimuli (Zheng, 2013), aberrant activity of TRP channels likely initiates and/or propagates ND processes, especially cell death, via increased intracellular Ca^2+^ in various brain regions (Moran, 2018; Hong et al., 2020; Huang et al., 2020). Here, we focus on the function of TRP channels associated with Ca^2+^ signaling in neurons and glial cells (Figure 1A) (Nilius, 2007; Bollimuntha et al., 2011; Zheng, 2013; Zhang and Liao, 2015; Jardin et al., 2017; Sawamura et al., 2017; Hasan and Zhang, 2018; Samanta et al., 2018; Cornillot et al., 2019; Enders et al., 2020; Wang et al., 2020). Based on sequence homology, the TRP family currently comprises 28 mammalian channels and is subdivided into six subfamilies: TRP canonical (TRPC), TRP vanilloid (TRPV), TRP ankyrin (TRPA), TRP melastatin (TRPM), TRP polycystin (TRPP), and TRP mucolipin (TRPML) (Nilius, 2007; Selvaraj et al., 2010; Nishida et al., 2015; Sawamura et al., 2017). Most TRP channels are non-selective channels with consistent Ca^2+^ permeability (Samanta et al., 2018) and each TRP subtype responds to various temperatures, ligands, as well as specific agonists and activators (Figure 1B) (Luo et al., 2020). TRP channels are tetramers formed by monomers that share a common structure comprising six transmembrane domains and containing cation-selective pores (Hellmich and Gaudet, 2014). Numerous studies have reported that these TRP channels are related to neuronal cell death that is associated with abnormal Ca^2+^ homeostasis (Gees et al., 2010; Sawamura et al., 2017).

### TRPC (Classic or Canonical)

TRPC was the first TRP group identified in mammals (Selvaraj et al., 2010). The TRPC subfamily contains members: TRPC1-7 (Wang et al., 2020). With the exception of TRPC2, all TRPC channels are widely expressed in the brain from the embryonic period to adulthood (Douglas et al., 2003). TRPC channels can form functional channels by heteromeric interactions, functioning as non-selective Ca^2+^ entry channels with distinct activation modes (Villereal, 2006). Thus, TRPC channels play an important role in regulating basic neuronal processes. TRPC1 is highly expressed and involved in the early development and proliferation of neurons (Yamamoto et al., 2005; Hentschke et al., 2006) as well as synaptic transmission (Broker-Lai et al., 2017; Wang et al., 2020). TRPC1 and TRPC4 have been reported to regulate neuronal cell death in response to seizures in the hippocampus and septum (Broker-Lai et al., 2017). The TRPC1/4/5 channel has been expressed in the somatosensory cortex, hippocampus, and motor cortex of adult rats (Riccio et al., 2002; Moran et al., 2004; Fowler et al., 2007). In particular, the dense expression of TRPC3 regulates hippocampal neuronal excitability and memory function (Neuner et al., 2015). The abnormal increase in sustained cytosolic Ca^2+^ by TRPC5 activation causes neuronal damage through the calpain-caspase-dependent pathway and the CaM kinase as seen in HD (Hong et al., 2015). Spinocerebellar ataxia type 14 (SCA14) is an autosomal dominant ND caused by a mutation in protein kinase Cγ (Wong et al., 2018). This mutation of SCA14 has been demonstrated to cause phosphorylation failure in TRPC3 channels, resulting in persistent Ca^2+^ entry that may contribute to neurodegeneration (Adachi et al., 2008). On the other hand, TRPC3 or TRPC6 promotes neurotrophin action on brain-derived neurotrophic factor (BDNF) by improving neuronal survival through Ca^2+^ influx (Huang et al., 2011). All TRPC channels are expressed in astrocytes and TRPC1 and TRPC3 play a critical role in astrocyte store-operated Ca^2+^ entry, which is induced by endoplasmic reticulum depletion (Verkhratsky et al., 2014). TRPC1 and TRPC6 are also expressed in rat microglia (Zhang and Liao, 2015). Thus, some TRPC channels exhibit different functions in normal physiological or pathological events, depending on Ca^2+^ signaling in the brain (Huang et al., 2011; Li et al., 2012; Neuner et al., 2015).

### TRPM (Melastatin)

Of all TRP channels, the TRPM subfamily has the largest and most diverse expression levels and has been strongly implicated in NDs (Samanta et al., 2018). The TRPM channel consists of eight members (TRPM1-8) and shares common structural characteristics with other TRP channels (Huang et al., 2020). However, they have a variety of C-terminal sections with active enzyme domains and a unique N-terminal without ankyrin repeats involved in channel assembly and trafficking (Huang et al., 2020). A distinctive feature of TRPM channels is the regulation of Ca^2+^ and magnesium (Mg^2+^) homeostasis, and TRPM (2–7) are mainly expressed in the CNS. In addition, TRPM2 is activated by a wide range of factors including NAD^+^-related metabolites, adenosine diphosphate-ribose, oxidative stress, and depletion of glutathione (GSH) (Sita et al., 2018). Increased levels of reactive oxygen species (ROS) due to GSH depletion causes TRPM2-dependent Ca^2+^ influx to induce neuronal cell death, suggesting that several neurological disorders, including AD, PD, and bipolar disorder (Akyuva and Naziroglu, 2020). In addition, an increase in intracellular Ca^2+^ and Aβ induced by TRPM2 activity induces neuronal cell death in the rat striatum (Belrose and Jackson, 2018). Mg^2+^ is the second most abundant cation and essential cofactor in various enzymatic reactions (Ryazanova et al., 2010). TRPM2 is expressed by both microglia and astrocytes, which regulate gliosis and immune cell function (Wang et al., 2016; Huang et al., 2017). TRPM7 is permeable to Mg^2+^ and maintains Mg^2+^ homeostasis (Ryazanova et al., 2010). In mouse cortical neurons, inhibition of TRPM7 expression protects against neuronal cell damage (Asrar and Aarts, 2013; Huang et al., 2020). TRPM7 is also found in astrocytes and microglia to control migration, proliferation, and invasion (Siddiqui et al., 2014; Zeng et al., 2015).

### TRPV (Vanilloid)

TRPV channels form homo- or heterotetrameric complexes and are non-selective cation channels (Startek et al., 2019). The TRPV subfamily consists of six members (TRPV1-6) that are located mostly on the plasma membrane (Zhai et al., 2020). Recent studies on pathological TRPV1 expression in the brain have been performed (Mickle et al., 2015). TRPV1 activation induces caspase-3 dependent programmed cell death through Ca^2+^-mediated signaling, resulting in cell death of cortical neurons (Ho et al., 2012; Song et al., 2013) and also triggers cell death through L-type Ca^2+^ channels and Ca^2+^ influx in rat cortical neurons (Shirakawa et al., 2008). The activation of cannabinoid 1 (CB1) receptors stimulates TRPV1 activity, leading to increased intracellular Ca^2+^ and cell death of mesencephalic dopaminergic neurons (Kim et al., 2005, 2008). TRPV1 activation induces apoptotic cell death in rat cortical neurons, leading to chronic epilepsy distinguished by abnormal brain activity (Fu et al., 2009). TRPV1 activation in microglia plays a positive role in promoting microglial phagocytosis in damaged cells while disrupting mitochondria and increasing ROS production (Kim et al., 2006; Hassan et al., 2014). TRPV1 has been shown to affect the migration of astrocytes (Ho et al., 2014). Abnormal function of TRPV4 leads to neuronal dysfunction and axonal degeneration due to increased Ca^2+^ via Ca^2+^/calmodulin-dependent protein kinase II (CaMKII) (Woolums et al., 2020). TRPV4 plays a role in regulating the osmotic pressure in the brain and is highly expressed throughout glial cells associated with ND (Liedtke and Friedman, 2003; Rakers et al., 2017). Thus, these channels play an important role in Ca^2+^ homeostasis and are therapeutic targets for various disorders.

### TRPA (Ankyrin)

TRPA1 was first identified as an ankyrin-like transmembrane protein and the solitary member of the mammalian TRPA subfamily (Yang and Li, 2016). TRPA1 is a non-selective cation channel formed by homo- or heterotetramer subunits with a cytosolic N-terminal domain (16 ankyrin repeat sequence) and C-terminal Ca^2+^-binding domains (Nilius et al., 2011; Fernandes et al., 2012). The TRPA1 channel responds to a variety of ligands, such as temperature, osmotic changes, and endogenous compounds (Nishida et al., 2015). To date, the reported role of TRPA1 in neurons is the mediation of pain, cold, inflammation, and itch sensation (Fernandes et al., 2012). Recent reports indicate that TRPA1 hyperactivation causes Aβ oligomer-mediated rapid Ca^2+^ signaling (Bosson et al., 2017; Hong et al., 2020). Additionally, ablation of TRPA1 in APP/PS1 transgenic mice attenuated the progression of AD, improved learning and memory conditions, and reduced Aβ plaques and cytokines (Lee et al., 2016). Similarly, TRPA1 channels promote Ca^2+^ hyperactivity of astrocytes and then contribute to synaptic dysfunction due to the oligomeric forms of Aβ peptide (Lee et al., 2016; Bosson et al., 2017; Logashina et al., 2019; Alavi et al., 2020). In addition, TRPA1 mediates Ca^2+^ signaling in astrocytes, resulting in dysregulation of synaptic activity in AD (Bosson et al., 2017).

### Other Channels

TRPML and TRPP have limited similarity to other TRP family members (Samanta et al., 2018; Huang et al., 2020). TRPML channels (TRPML1-3) are Ca^2+^ permeable cation channels that each contain six transmembrane segments with helices (S1–S6) and a pore site comprised of S5, S6, and two pore helices (PH1 and PH2) (Schmiege et al., 2018; Tedeschi et al., 2019). TRPML channels are mostly located in intracellular compartments instead of the plasma membrane (Clement et al., 2020). TRPP channels share high protein sequence similarity with TRPML channels and are located in the primary cilia consisting of TRPP1 (also known as PKD1) and TRPP2 (PKD2) (Samanta et al., 2018). To date, evidence indicates that various TRP channels are expressed in the CNS and play important roles in the development of several NDs (Sawamura et al., 2017; Samanta et al., 2018). In particular, TRP channels and Ca^2+^ homeostasis (Bezprozvanny, 2010) are likely to underpin Ca^2+^-dependent neuronal death in NDs (Sawamura et al., 2017; Hong et al., 2020).

## Pathophysiological Role of Plasma Membrane Calcium ATPases

Of the various proteins involved in Ca^2+^ signaling, PMCA is the most sensitive Ca^2+^ detector that regulates Ca^2+^ homeostasis (Boczek et al., 2019). PMCA exists in four known isoforms (Boczek et al., 2019). In both mice and humans, PMCAs 1–4 exhibit anatomically distinct expression patterns, such that isoforms 1 and 4 are ubiquitously expressed in all tissue types, whereas PMCA2 and PMCA3 are tissue-specific and exclusive in neurons of the brain (Kip et al., 2006). In addition, PMCA1, 2, and 4 were detected in rat cortical astrocytes (Fresu et al., 1999) (Table 2). The general structure of PMCA consists of 10 transmembrane domains (TM) with the N- and C-terminal ends on the cytosolic side (Stafford et al., 2017). The physiological functions of PMCA include the regulation and maintenance of optimal Ca^2+^ homeostasis (Bagur and Hajnoczky, 2017). PMCA is an ATP-driven Ca^2+^ pump that maintains low resting intracellular Ca^2+^ concentration ([Ca^2+^]i) to prevent cytotoxic Ca^2+^overload-mediated cell death through activation of ion channels such as TRP (Zundorf and Reiser, 2011). In addition, PMCA is involved in Ca^2+^-induced intracellular acidification by countertransport of H^+^ ions (Vale-Gonzalez et al., 2006; Majdi et al., 2016). Thus, PMCA plays a vital role in controlling cell survival and cell death (Stafford et al., 2017). PMCA expression changes significantly during brain development (Boczek et al., 2019). One of the characteristics of brain aging is a Ca^2+^ homeostasis disorder, which can result in detrimental consequences on neuronal function (Boczek et al., 2019). Overall, PMCAs have been attributed a housekeeping role in maintaining intracellular Ca^2+^ levels through precise regulation of Ca^2+^ homeostasis (Strehler et al., 2007). However, the altered composition of PMCA is associated with a less efficient Ca^2+^ extrusion system, increasing the risk of neurodegenerative processes (Strehler and Thayer, 2018). *ATP2B2* is a deafness-associated gene that encodes PMCA2 (Smits et al., 2019). A recent study reported a link between PMCA2 and autism spectrum disorder (ASD) (Yang et al., 2013). ASD is a group of neurodevelopmental disorders that results in deficits in social interaction (Chaste and Leboyer, 2012; Fatemi et al., 2012). Intracellular Ca^2+^ levels are crucial for regulating neuronal survival, differentiation, and migration (Bezprozvanny, 2010). Perturbations in these processes underlie the pathogenesis of autism spectrum disorders (Gilbert and Man, 2017). *ATP2B3* mutations are associated with X-linked cerebellar ataxia and Ca^2+^ extrusion disorders in patients with cerebellar ataxia and developmental delay (Zanni et al., 2012; Mazzitelli and Adamo, 2014; Cali et al., 2015). Several neurotoxic agents, such as oxidation and age, downregulate PMCA function and increase susceptibility to NDs (Zaidi, 2010). In particular, the internalization of PMCA2 initiated by protease function in rat hippocampal pyramidal cells after glutamate exposure or kainate-induced seizures, in which loss of PMCA function occurs, may contribute to Ca^2+^ dysregulation and lead to neuronal cell death (Pottorf et al., 2006; Stafford et al., 2017). A decrease in PMCA activity and increased Ca^2+^ may cause cell death depending on the degree of cytosolic accumulation of tau and Aβ in AD (Boczek et al., 2019). In addition, PMCA expression is decreased in the cortex of postmortem brains of patients with AD (Berrocal et al., 2019; Boczek et al., 2019).

**Table 2:** A summary of the transient receptor potential (TRP) subtypes found in distribution of central nervous system (CNS) cell types.

**PMCA subfamily**	**Expression in brain**	**Expression in glia**	**Disorders**	**References**
PMCA1	- Ubiquitous in brain (human and rat). - Cerebellum, cerebral cortex, brain stem (Human)	Rat cortical astrocytes	AD, PD	Stauffer et al., 1995; Fresu et al., 1999; Brini et al., 2013
PMCA2	- Cerebellar purkinje neurons (human/mouse) - cerebellum, cerebral cortex, brain stem (Human)	Rat cortical astrocytes	AD, PD, cerebellar ataxias, sensory neuron diseases	Stauffer et al., 1995; Fresu et al., 1999; Kurnellas et al., 2007; Empson et al., 2010; Hajieva et al., 2018; Strehler and Thayer, 2018
PMCA3	- Cerebellum, cerebral cortex (Human) - Cerebellum and hippocampus (Rat)	Limited	Cerebellar ataxias, sensory neuron diseases	Stauffer et al., 1995; Zanni et al., 2012; Strehler and Thayer, 2018
PMCA4	- Ubiquitous in brain (human/rat) - Cerebellum, cerebral cortex, brain stem (Human)	Rat cortical astrocytes	AD, PD	Stauffer et al., 1995; Fresu et al., 1999; Brini et al., 2013; Zaidi et al., 2018

*PMCA, plasma membrane Ca^2+^ ATPase; AD, Alzheimer's disease; PD, Parkinson's disease*.

## pH Regulation by PMCA in Neurodegenerative Diseases

As mentioned above, PMCAs have a Ca^2+^ extrusion function on the membrane and another important function, namely H^+^ uptake (Stafford et al., 2017). Since PMCA is responsible for control of Ca^2+^ extrusion and H^+^ uptake rates, it provides an important link between Ca^2+^ signaling and intracellular pH (pH_i_) in neurons (Hwang et al., 2011). Mechanisms that maintain strict pH homeostasis in the brain control neuronal excitability, synaptic transmission, neurotransmitter uptake, nociception, and inflammation (Chesler, 2003; Dhaka et al., 2009; Casey et al., 2010; Hwang et al., 2011). Changes in pH caused via pH-sensitive or pH-regulated ion channels are detrimental to brain function and can cause multiple degenerative diseases (Ruffin et al., 2014). Neuronal excitability is particularly sensitive to changes in intracellular and extracellular pH mediated by various ion channels (Parker and Boron, 2013). The activation of TRPV1 has been reported to induce a rise in Ca^2+^ and cause intracellular acidification via the activation of PMCA in the rat trigeminal ganglion (Hwang et al., 2011). Under normal conditions, acidification conditions are promptly returned to and maintained at normal pH levels through a physiological pH_i_ recovery mechanism involving the regulation of Na^+^/H^+^ exchangers (NHE) and Na^+^-${\text{HCO}}_{3}^{-}$ cotransporter (NBCs) in the brain (Chesler, 2003; Sinning and Hubner, 2013; Ruffin et al., 2014; Bose et al., 2015). NHE1 is abundantly expressed in all neuronal cells and astrocytes, regulating cell volume homeostasis and pH_i_ (Song et al., 2020). NBC1 is also widely expressed in astrocytes throughout the brain (Annunziato et al., 2013) (Figure 1A). However, functional inhibition of pH_i_ recovery mechanism in pathological conditions leads to excessive intracellular acidification (Majdi et al., 2016). Therefore, although the exact underlying mechanism that causes intracellular acidification in brain neurons is unknown. However, it appears that persistent intracellular acidification condition promotes irreversible neuronal damage and induces amyloid aggregation in the brains of patients with AD (Xiong et al., 2008; Ruffin et al., 2014).

## Conclusion

Intracellular Ca^2+^ and pH regulation play vital roles in both physiological and pathological conditions. Abnormal changes in Ca^2+^ or pH typically cause cell death. TRP channels are involved in Ca^2+^ influx, which affects neuronal and glial functions under normal physiological conditions. However, altered expression of TRP channels can lead to excess Ca^2+^ influx, and intracellular Ca^2+^ overload is maintained due to *ATP2B2*, oxidation, and aging-related downregulation of PMCA, leading to Ca^2+^-dependent cell death. Alternatively, overexpression of PMCA due to cytoplasmic Ca^2+^ overload causes continuous acidification from inhibition of the pH_i_ recovery mechanisms by oxidative stress or programmed cell death, resulting in acidification-dependent cell death (Figure 2) (Harguindey et al., 2017, 2019). To date, TRP channels have been investigated for their role in NDs. However, targeting TRP channels and PMCA, including Ca^2+^ and pH regulation, as a treatment for NDs requires a deeper understanding of their function in both health and disease. This review describes potential therapeutic targets for NDs by discussing TRP channels and PMCA responsible for the disruption of intracellular Ca^2+^ and pH homeostasis that underpin ND development.

## Author Contributions

C-KP and YK conceived and supervised the project. S-MH, JL, C-KP, and YK wrote the paper. All authors contributed to the article and approved the submitted version.

## Conflict of Interest

The authors declare that the research was conducted in the absence of any commercial or financial relationships that could be construed as a potential conflict of interest.
